# Cognigram^TM^ Computerized Cognitive Testing: Longitudinal Validation Study Over Four Years

**DOI:** 10.1002/alz70856_106669

**Published:** 2026-01-08

**Authors:** Andrew R Frank, Laura Ault, Karra D Harrington, Jason Cromer, Bruce Wallace, Lisa Sweet, Neil W. Thomas, Michael Breau, Rafik Goubran, Frank Knoefel

**Affiliations:** ^1^ University of Ottawa, Ottawa, ON, Canada; ^2^ Bruyere Health Research Institute, Ottawa, ON, Canada; ^3^ Cogstate, New Haven, CT, USA; ^4^ Yale University, New Haven, CT, USA; ^5^ Carleton University, Ottawa, ON, Canada; ^6^ Bruyere Health, Ottawa, ON, Canada

## Abstract

**Background:**

As the population ages, there is increasing need for biomarkers which can predict if individuals will worsen cognitively or remain stable. Cognigram™ (CG) is a computerized cognitive battery which uses card‐sorting tasks to assess cognitive abilities, thus acting as a “digital biomarker”, which may predict cognitive decline earlier than paper‐based cognitive tests.

**Method:**

Participants with clinically‐diagnosed mild cognitive impairment (MCI) were asked to complete CG testing and traditional neuropsychological testing over a period of four years. Individuals who demonstrated a significant deterioration on cognitive and functional testing over time (as determined by blinded expert consensus conference) were labelled as “decliners”. Those who did not deteriorate on testing were labelled as “non‐decliners”. CG findings were analyzed for early predictive changes which could differentiate between decliners and non‐decliners.

**Result:**

Seventeen (M=12, F=5) individuals were identified for this analysis, and were classified over an average of 34 months in the study (range 6‐61 months) as decliners (*n* = 8, 37.5% female, average age 75.6) or non‐decliners (*n* = 9, 22.2% female, average age 75.4). Over the first year, the CG one‐back task (“Is this card the same as the previous card?”) consistently showed the decliner group having more negative change from baseline at each timepoint compared to the non‐decliner group, with moderate effect sizes observed at 3 and 9 months and a very large effect size observed at 12 months (Hedges’ g=‐1.54, *p* = 0.03).

**Conclusion:**

The CG one‐back task showed greater negative change over one year in participants observed to decline clinically during the study. A larger study is needed to determine if CG can predict the likelihood of long‐term cognitive decline in patients with mild cognitive impairment.

